# The Effect of Esmolol on Tissue Perfusion and Clinical Prognosis of Patients with Severe Sepsis: A Prospective Cohort Study

**DOI:** 10.1155/2016/1038034

**Published:** 2016-08-29

**Authors:** Xiuling Shang, Kaiyu Wang, Jingqing Xu, Shurong Gong, Yong Ye, Kaihua Chen, Fayang Lian, Wei Chen, Rongguo Yu

**Affiliations:** ^1^Department of Critical Care Medicine, Fujian Provincial Clinical College of Fujian Medical University, Fujian Provincial Hospital, Fuzhou, Fujian 350001, China; ^2^Department of Research, Fujian Provincial Hospital, Fuzhou, Fujian 350001, China; ^3^Department of Critical Care Medicine, Chinese Medicine Hospital, Fuzhou, Fujian 350001, China

## Abstract

* Purpose*. This study was aimed at investigating the effect of esmolol on tissue perfusion and the clinical prognosis of patients with severe sepsis.* Materials and Methods*. One hundred fifty-one patients with severe sepsis were selected and divided into the esmolol group (*n* = 75) or the control group (*n* = 76), who received conventional antiseptic shock treatment. The esmolol group received a continuous infusion of esmolol via a central venous catheter, and their heart rate (HR) was maintained at 70–100 bpm over 72 hours.* Results*. The HR of all patients reached the target level within 72 hours of treatment for both groups. The effect of esmolol on PvaCO_2_ was only significant at 48 hours (*P* < 0.05). ScvO_2_ increased in the esmolol group and decreased in the control group (*P* < 0.01). Lac showed a linear downward trend over the treatment time, but the reduction was more significant in the control group at 48 hours (*P* < 0.05) between the two groups. Kaplan-Meier analysis showed a significantly shorter duration of mechanical ventilation in the esmolol group than in the control group (*P* < 0.05).* Conclusions*. Esmolol reduced the duration of mechanical ventilation in patients with severe sepsis, with no significant effect on circulatory function or tissue perfusion.

## 1. Introduction

Sepsis is a systemic inflammatory response syndrome due to suspected or confirmed infection and can develop into severe sepsis, septic shock, and multiple organ dysfunction syndrome (MODS) [1]. Excessive activation of the sympathetic nervous system and a substantial increase in catecholamine secretion are important causes of cardiac dysfunction in patients with serious infections and septic shock; thus, the suppression of sympathetic nerve activation is a novel target for treating sepsis. Previously, researchers believed that *β*-blockers had negative inotropic effects and lowered blood pressure and thus were generally not suitable for treating septic shock. However, an increasing body of recent clinical and basic studies has shown that, for patients with septic shock, *β*-blockers not only effectively control HR but also protect cardiac function and improve the clinical prognosis. This study aimed to investigate the effect of esmolol on the hemodynamics, tissue perfusion, and clinical prognosis of patients with severe sepsis and to explore the value of esmolol in the clinic.

## 2. Materials and Methods

### 2.1. Clinical Information

We conducted a prospective cohort clinical trial. One hundred ninety patients with severe sepsis were treated in the Department of Critical Care Medicine (Intensive Care Unit [ICU]), Fujian Provincial Hospital, from January 2010 to January 2013. Of these patients, after esmolol treatment, three patients had a decreased HR (<70 bpm), two patients had severe arrhythmia, four patients had low blood pressure, 21 patients were hospitalized for less than 72 hours because of rapid progression of the condition, and nine patients and their families declined to participate in this study. Therefore, these patients were excluded from this study. Accordingly, 151 patients met the inclusion criteria and were enrolled as study subjects. These patients were assigned into the esmolol group (*n* = 75) or the control group (*n* = 76) according to esmolol usage. There were 107 male patients and 44 female patients.

### 2.2. Inclusion Criteria

Inclusion criteria were as follows: (1) age > 18 years; (2) severe sepsis (all infections were eligible, including pneumonia, peritonitis, and intracranial infection) diagnosis according to the* Campaign to Save Septic Patients: 2008 Treatment Guidelines for Severe Sepsis and Septic Shock*; (3) mechanical ventilation via endotracheal intubation with a tidal volume of 6 mL/kg; and (4) satisfactory sedation and analgesic treatment, with HR > 100 bpm.

### 2.3. Exclusion Criteria

Exclusion criteria were as follows: (1) preexisting cardiac dysfunction, valvular heart disease, high-degree atrioventricular block; (2) acute or chronic pulmonary heart disease; (3) history of serious asthma; (4) chronic renal insufficiency; (5) cancer, autoimmune diseases, or contraindications for deep venous catheter placement; and (6) insulin-dependent diabetes.

This study complied with medical ethics standards and obtained approval from the Ethics Committee of our hospital (K2010-001-01). Moreover, because all the patients were intubated, we obtained informed consent from the patients' family members, who signed the informed consent form before the study.

### 2.4. Groups and Treatment

The 151 patients with severe sepsis were assigned into the esmolol group or the control group. All of the patients continued to receive routine treatment, including anti-infective treatment, respiratory and circulatory support, sedation and analgesic treatment, and nutritional support. In addition, patients in the esmolol group received a continuous infusion of esmolol via a micropump through a catheter placed in the superior vena cava. The initial dose was 0.05 mg/kg/min and was adjusted based on heart rate (HR) (target HR: 70 bpm < HR < 100 bpm within 72 hours). In case of low blood pressure, norepinephrine or dopamine was adjusted as necessary to maintain a mean arterial pressure (MAP) ≥ 65 mmHg. The infusion rate was adjusted based on the central venous pressure (CVP) to maintain the CVP at 10 to 15 mmHg. The control group also received natural saline via a micropump; in the same way, the esmolol group received esmolol.

### 2.5. Measures

We collected data on hemodynamic metrics (MAP, CVP, and HR), tissue perfusion indicators (central venous oxygen saturation [ScvO_2_], venous-arterial carbon dioxide partial pressure [P(va)CO_2_], and arterial blood lactate [Lac]), vasoactive-inotropic score (IS) [2], and fluid intake before and after 24 hours, 48 hours, and 72 hours of treatment in the two groups. The duration of the ICU stay (days) and the duration of mechanical ventilation (days) were also recorded.

 IS = dopamine (mcg/kg/min) + dobutamine (mcg/kg/min) + 100 × epinephrine (mcg/kg/min) + 100 norepinephrine (mcg/kg/min).

### 2.6. Statistical Analysis

SPSS 19.0 software was used for the statistical analysis. Measurement data are expressed as the mean ± standard deviation, and a two-independent-sample *t*-test was performed for group comparisons. One-way analysis of variance with repeated measures was performed to analyze changes in continuous variables from baseline between the two groups. A chi-square test was performed to analyze the 28-day mortality rate. A log-rank test was performed to analyze the ICU stay and the duration of mechanical ventilation. *P* < 0.05 was considered statistically significant.

## 3. Results

*Group Comparison of Baseline Data*. No significant difference was observed between the two groups at study entry with respect to age (Table 1), gender, Acute Physiology and Chronic Health Evaluation II (APACHE-II) score, hemodynamics (MAP, CVP, and HR), tissue perfusion indicators (ScvO_2_, P(va)CO_2_, and Lac), or vasoactive-inotropic score (IS) (Table 2) (all *P* > 0.05), suggesting that the baseline data were balanced and comparable between the two groups.

Fluid intake was significantly lower in the esmolol group than in the control group at 24 hours, 48 hours, and 72 hours after treatment (all *P* < 0.01). After treatment, the HR trended downward in both groups, with a significant difference between the two groups at all time-points (all *P* < 0.01). For the esmolol group, the HR of all the patients reached the target level within 72 hours of treatment. No significant difference between the two groups was observed for MAP, CVP, and IS at any time-point (all *P* > 0.05), as shown in Table 3.

For both groups, compared with the baseline measurement, a significant change was observed in Lac and PvaCO_2_ at 24 hours, 48 hours, and 72 hours after treatment (all *P* < 0.05); however, no significant change was observed in ScvO_2_ after treatment (*P* > 0.05). In both groups, compared with the baseline, PvaCO_2_ showed a linear downward trend over the treatment time, and the effect of esmolol on PvaCO_2_ was only significant at 48 hours (*P* < 0.05), with no difference in the other time-points (*P* > 0.05). ScvO_2_ increased over the treatment time in the esmolol group and decreased over the treatment time in the control group (*P* < 0.01). Lac showed a linear downward trend over the treatment time, but the reduction was more significant in the control group at 48 hours (*P* < 0.05), with no difference in the other time-points between the two groups (*P* > 0.05), as illustrated in Table 4.

One-way analysis of variance with repeated measures between the two groups (Table 5) (significance level: *α* = 0.05) showed a significant difference in PvaCO_2_ (*P* < 0.005), HR (*P* < 0.001), ScvO_2_ (*P* < 0.001), and fluid intake (*P* < 0.001). No significant difference was observed for MAP, CVP, IS, or Lac between the groups (all *P* > 0.05).

**Figure 1:**
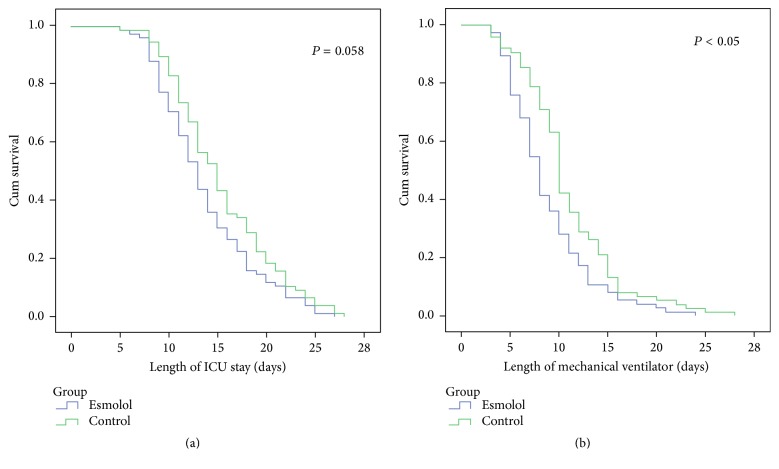
The survival analysis of length of ICU stay (a) and length of mechanical ventilation (b) based on the Kaplan-Meier analysis.

The group comparison of prognostic indicators (Table 6) indicated that no significant difference was observed in the 28-day mortality rate between the two groups (5.3% [esmolol group] versus 7.9% [control group], *P* = 0.760). A rank sum test showed a significant difference between the two groups with respect to ICU stay (*P* = 0.035) and duration of mechanical ventilation (*P* = 0.002). Survival analysis (Kaplan-Meier analysis) showed no significant difference between the two groups in length of ICU stay (*P* = 0.058), as shown in Figure 1(a). However, the duration of mechanical ventilation was significantly shorter in the esmolol group than in the control group (*P* < 0.05), as shown in Figure 1(b).

## 4. Discussion

*β*-Blockers have been investigated in studies of sepsis treatment for five decades. In the 1960s, Berk et al. [3] used a dog model to demonstrate that excessive *β*-adrenergic stimulation induced sepsis and that propranolol improved blood pressure and blood pH; this treatment even improved survival. Suzuki et al. [4] used a septic mouse model to inject esmolol, a selective *β*1-adrenoreceptor antagonist, and found that esmolol reduced the HR, blood pressure, and serum TNF-*α* levels in the experimental group. No significant effect on the Lac level was observed, suggesting that esmolol did not increase oxygen consumption in tissues. *β*-Blockers significantly reduce HR and time-course HR variability, thereby harmonizing cardiac force and frequency and reducing myocardial oxygen consumption [5]. Moreover, *β*-blockers reduce the expression of chemokines and inflammatory cytokines during cardiac dysfunction [6] and play a cardioprotective role in acute myocardial stunning [7]. With respect to septic shock, *β*-blockers also stabilize the circulation and improve myocardial injury [8, 9]. Therefore, *β*-blockers play an important role and show promise for the treatment of sepsis.

In contrast with experimental animals and basic research, which have demonstrated promise for *β*-blockers, clinical trials and studies of *β*-blockers for sepsis have produced inconsistent results. Schmittinger et al. [10] studied 40 patients with septic shock who required fluid resuscitation and vasoactive drugs (including norepinephrine, milrinone, and vasopressin). This study found that, after metoprolol treatment, in 39 patients, the HR was controlled at 65–95 bpm with no MAP decrease, the stroke volume index (SVI) increased, the cardiac index remained stable, and the Lac level decreased significantly. The present study showed that after 72 hours of esmolol treatment, the HR was controlled at ≤100 bpm with a significant decrease in MAP, no significant change in CVP, and no significant increase in IS. These results suggest that for patients with severe sepsis, esmolol is effective in controlling HR, with no decrease in blood pressure. Moreover, no increase in the dose of vasoactive drugs is required. Therefore, esmolol is safe and feasible in clinical practice.

Small clinical studies and recent large retrospective studies have shown favorable results for the application of *β*-blockers. Christensen et al. [11] conducted a cohort study to analyze 8,087 adult cases aged ≥45 years who were treated from 1999 to 2005. The results showed that for patients who were on long-term *β*-blockers (>125 days) before ICU admission (*β*-blocker group), the 30-day mortality rate after ICU admission was 25.7%, which was significantly lower than that of the control group (31.4%) (odds ratio [OR] 0.74 and 95% confidence interval [CI] 0.63–0.87; *P* < 0.05). These results suggest that the use of *β*-blockers before ICU admission reduced mortality. Italian researchers Morelli et al. [12] conducted a randomized controlled clinical trial of 154 patients with septic shock. The patients were randomly assigned into one of two groups: one receiving continuous intravenous infusion of esmolol to maintain the HR at 80 to 94 bpm and another (control group) receiving routine treatment. The results showed that in the esmolol group, the HR of all of the patients was controlled within the target range; these results are consistent with those of the present study. Moreover, in the study by Morelli et al., the 28-day mortality rate was 49.4% in the esmolol group and 80.5% in the control group (OR 0.39, 95% CI 0.26–0.59; *P* < 0.001). Therefore, these authors concluded that esmolol effectively controlled the HR and improved the survival of patients with sepsis. In another study, Morelli et al. [13] found that esmolol favorably controlled the HR of patients with septic shock without increasing Lac and mixed SvO_2_, thereby having no adverse effect on tissue perfusion. This study also showed no significant difference in Lac reduction between the two groups (*P* > 0.05); thus, we conclude that esmolol has no effect on tissue perfusion in patients with sepsis.

Shock, in essence, is tissue ischemia and hypoxia; thus, to treat septic shock, it is important to implement active resuscitation and to improve tissue perfusion while administering anti-infective treatment. The presence of Lac is a delayed manifestation of tissue hypoperfusion, and ScvO_2_ and P(va)CO_2_ are two early clinical indicators of tissue perfusion. Studies have shown that P(va)CO_2_ and ScvO_2_ are related [14–16]. In the present study, one-way analysis of variance with repeated measures showed that Lac was slowly cleared in both groups, with no significant difference between the two groups. Morelli et al. [13] found that esmolol had no effect on SV and significantly increased the microcirculation in patients with sepsis. The present study also found that PvaCO_2_ decreased over time in the esmolol group; thus, we believe that esmolol increases circulation, which is consistent with previous research. Moreover, ScvO_2_ increased slightly in the esmolol group, with a significant difference between the two groups. However, more research is necessary to verify whether this effect is due to an esmolol-induced reduction in tissue oxygen consumption. Furthermore, we found that, in the esmolol group, fluid intake was reduced, with no significant changes in IS and CVP. Esmolol, therefore, reduced the adverse effects of excessive fluid intake. It was also observed that the HR was reduced in the esmolol group compared with the control group, with no significant change in MAP, suggesting that esmolol effectively controlled HR within the target range, without significant effects on cardiac systolic function. The duration of mechanical ventilation was shortened in the experimental group, an effect that was likely related to reduced HR, fluid intake, and pulmonary fluid. The 28-day mortality rate was 5.3% in the esmolol group and 7.9% in the control group, with no significant difference between the two groups (*P* = 0.760). No significant difference in ICU stay was observed between the two groups, likely due to the small sample size (this being a single-center study) and bias in the exclusion criteria.

Multiple basic and clinical studies of severe sepsis have shown a positive outlook for *β*-blockers. In particular, the cardioprotective effect and clinical outcomes of ultra-short-acting *β*-4-blockers in patients with septic shock are especially encouraging. Nevertheless, many questions remain unanswered, such as the timing of the treatment and dosage, potential synergistic effects between different types of *β*-blockers, and the relationship between the efficacy of *β*-blockers and the type of pathogens and infected sites. Hence, large clinical studies are necessary.

## 5. Limitation

This clinical trial lacks the measurement of tissue bacterial growth and of cytokines such as tumor necrosis factor alpha, as in the experimental study by Dimopoulos et al. [17]. This fact constitutes the limitation of the present investigation.

## 6. Conclusion

In summary, esmolol, an ultra-short-acting *β*-blocker, significantly controlled HR and reduced the duration of mechanical ventilation in patients with severe sepsis, with no significant effect on circulatory function or tissue perfusion. The observed decrease in PvaCO_2_ may be related to the increased microcirculation associated with esmolol; the mechanism of increased ScvO_2_ is not clear and requires further research.

## Figures and Tables

**Table 1:** Rank sum test of age (median) between the two groups.

Base value	Esmolol group (*n* = 75)	Control group (*n* = 76)	M–W *U* value	*P*
Age, median, years	58 (41–66)	59 (43–69)	2701	0.579

**Table 2:** Chi-square test and *t*-test of the baseline characteristics of the patients (mean).

Base value	Esmolol group (*n* = 75)	Control group (*n* = 76)	*t*/*χ* ^2^	*P*
Male, *n* (%)	54 (72.0)	53 (69.7)	0.094	0.760
APACHE-II score, mean ± standard deviation	24.20 ± 7.66	25.46 ± 7.83	−0.999	0.319
HR, mean ± standard deviation	125.04 ± 13.28	127.21 ± 13.88	−0.982	0.328
MAP, mean ± standard deviation	74.71 ± 7.28	75.89 ± 6.61	−1.051	0.295
CVP, mean ± standard deviation	12.45 ± 3.16	11.99 ± 3.35	0.881	0.380
IS, mean ± standard deviation	10.99 ± 2.08	11.11 ± 1.81	−0.374	0.709
Lac, mean ± standard deviation	8.98 ± 3.09	9.74 ± 4.05	−1.301	0.195
P(va)CO_2_, mean ± standard deviation	9.54 ± 3.89	10.12 ± 3.52	−0.947	0.345
ScvO_2_, mean ± standard deviation	0.77 ± 0.06	0.78 ± 0.06	−0.537	0.592

Note: APACHE-II: Acute Physiology and Chronic Health Evaluation II; HR: heart rate; MAP: mean arterial pressure; CVP: central venous pressure; IS: vasoactive-inotropic score; Lac: arterial blood lactate; P(va)CO_2_: venous-arterial carbon dioxide partial pressure; ScvO_2_: central venous oxygen saturation.

**Table 3:** The effect of esmolol on hemodynamics in patients with severe sepsis.

(Time, H)	Esmolol group (*n* = 75) Mean ± standard deviation	Control group (*n* = 76) Mean ± standard deviation	*t*	*P*
*Fluid intake*				
24 h	4373.36 ± 571.86	4841.75 ± 658.89	−4.663	<0.01
48 h	4189.61 ± 515.52	4720.74 ± 648.60	−5.566	<0.01
72 h	3991.08 ± 486.73	4553.20 ± 591.72	−6.371	<0.01
*HR*				
Base value	125.04 ± 13.28	127.21 ± 13.88	−0.982	0.328
24 h	101.96 ± 7.36	110.12 ± 8.59	−6.272	<0.01
48 h	93.04 ± 4.52	102.57 ± 6.91	−10.039	<0.01
72 h	84.17 ± 6.26	94.47 ± 7.91	−8.861	<0.01
*MAP*				
Base value	74.71 ± 7.28	75.89 ± 6.61	−1.051	0.295
24 h	68.65 ± 9.72	68.54 ± 7.69	0.080	0.936
48 h	71.00 ± 11.80	68.39 ± 7.53	1.615	0.109
72 h	70.91 ± 10.57	68.14 ± 7.73	1.829	0.069
*CVP*				
Base value	12.45 ± 3.16	11.99 ± 3.35	0.881	0.380
24 h	10.04 ± 1.72	10.04 ± 1.71	0.002	0.998
48 h	10.08 ± 1.43	9.83 ± 1.23	1.158	0.249
72 h	9.77 ± 1.60	9.91 ± 1.64	−0.510	0.611
*IS*				
Base value	10.99 ± 2.08	11.11 ± 1.81	−0.374	0.709
24 h	10.92 ± 1.39	10.74 ± 1.40	0.794	0.428
48 h	9.56 ± 1.18	9.51 ± 1.44	0.243	0.808
72 h	9.09 ± 1.24	9.29 ± 1.23	−0.957	0.340

**Table 4:** The effect of esmolol on tissue perfusion indicators in patients with severe sepsis.

(Time, H)	Esmolol group (*n* = 75)	Control group (*n* = 76)	*t*	*P*
Mean ± standard deviation				
*Lac, mmol/L*				
Base value	8.98 ± 3.09	9.74 ± 4.05	−1.301	0.195
24	6.62 ± 2.43	6.82 ± 2.43	−0.522	0.603
48	3.87 ± 1.89	3.10 ± 1.9	2.493	0.014
72	2.32 ± 0.98	2.41 ± 1.07	−0.534	0.594
*P(va)CO*_*2*_ *, mmHg*				
Base value	9.54 ± 3.89	10.12 ± 3.52	−0.947	0.345
24	6.71 ± 3.29	7.26 ± 3.34	−1.019	0.310
48	5.11 ± 2.10	5.94 ± 2.38	−2.285	0.024
72	2.73 ± 1.08	3.06 ± 1.73	−1.415	0.160
*ScvO*_*2*_				
Base value	0.7711 ± 0.0566	0.7762 ± 0.0592	−0.537	0.592
24	0.8000 ± 0.0529	0.7679 ± 0.0510	3.798	0.000
48	0.7932 ± 0.0441	0.7657 ± 0.0569	3.318	0.001
72	0.7957 ± 0.0362	0.7636 ± 0.0551	4.227	0.000

**Table 5:** One-way analysis of variance with repeated measures between the two groups.

Mean ± standard deviation					
Group	Base value	24 h	48 h	72 h	*P*
*HR, bpm*					
Esmolol group	125.04 ± 13.28	101.96 ± 7.36	93.04 ± 4.52	84.17 ± 6.26	<0.001
Control group	127.21 ± 13.88	110.12 ± 8.59	102.57 ± 6.91	94.47 ± 7.91	
*MAP, mmHg*					
Esmolol group	74.71 ± 7.28	68.65 ± 9.72	71.00 ± 11.80	70.91 ± 10.57	0.277
Control group	75.89 ± 6.61	68.54 ± 7.69	68.39 ± 7.53	68.14 ± 7.73	
*CVP, mmHg*					
Esmolol group	12.45 ± 3.16	10.04 ± 1.72	10.08 ± 1.43	9.77 ± 1.60	0.385
Control group	11.99 ± 3.35	10.04 ± 1.71	9.83 ± 1.23	9.91 ± 1.64	
*IS*					
Esmolol group	10.99 ± 2.08	10.92 ± 1.39	9.56 ± 1.18	9.09 ± 1.24	0.881
Control group	11.11 ± 1.81	10.74 ± 1.40	9.51 ± 1.44	9.29 ± 1.23	
*Fluid intake, mL/24 h*					
Esmolol group		4373.36 ± 571.86	4189.61 ± 515.52	3991.08 ± 486.73	<0.001
Control group		4841.75 ± 658.89	4720.74 ± 648.60	4553.20 ± 591.72	
*Lac, mmol/L*					
Esmolol group	8.98 ± 3.09	6.62 ± 2.43	3.87 ± 1.89	2.32 ± 0.98	0.705
Control group	9.74 ± 4.05	6.82 ± 2.43	3.10 ± 1.90	2.401 ± 1.07	
*P(va)CO*_*2*_ *, mmHg*					
Esmolol group	9.54 ± 3.89	6.71 ± 3.29	5.11 ± 2.10	2.73 ± 1.08	0.017
Control group	10.12 ± 3.52	7.26 ± 3.34	5.94 ± 2.38	3.06 ± 1.73	
*ScvO*_*2*_					
Esmolol group	0.7712 ± 0.0566	0.8000 ± 0.0529	0.7932 ± 0.0441	0.7957 ± 0.0362	<0.001
Control group	0.7762 ± 0.0592	0.7679 ± 0.0510	0.7657 ± 0.0569	0.7636 ± 0.0551	

**Table 6:** Comparison of prognostic indicators between the two groups.

Result	Esmolol group (*n* = 75)	Control group (*n* = 76)	*χ* ^2^/M–W *U* value	*P*
28-day mortality rate, *n* (%)	4 (5.3)	6 (7.9)	0.093	0.760
ICU stay, median (d)	13 (10–17)	15 (11–19)	2285.5	0.035
Duration of mechanical ventilation, median (d)	8 (6–11)	10 (8–14)	2002.0	0.002
